# 室内灰尘中传统邻苯二甲酸酯及新型替代品的赋存特征、神经毒性风险与潜在机制

**DOI:** 10.3724/SP.J.1123.2025.06025

**Published:** 2026-05-08

**Authors:** Wei LI, Ke GAO, Kai HUA, Linxiao WANG, Wei WEI, Liping LU

**Affiliations:** 北京工业大学环境科学系区域大气复合污染防治北京市重点实验室，北京 100124; Key Laboratory of Beijing on Regional Air Pollution Control，Department of Environmental Science，Beijing University of Technology，Beijing 100124，China

**Keywords:** 邻苯二甲酸酯, 新型替代品, 室内灰尘, 赋存特征, 神经毒性风险, 潜在机制, phthalate esters, novel alternatives, indoor dust, occurrence characteristics, neurotoxicity risk, potential mechanisms

## Abstract

传统邻苯二甲酸酯（PAEs）及其新型替代品作为常用增塑剂，易从材料中迁移并富集于室内灰尘，对人体健康构成潜在风险。尽管其致癌与生殖毒性已被广泛研究，但其神经毒性，尤其是新型替代品的作用仍显不足。为此，本研究分析了校园典型微环境室内灰尘中目标化合物的污染特征，结合灰尘的经口摄入、吸入和皮肤接触3种暴露途径，以及吸收、分布、代谢、排泄和毒性模型，系统评估了不同人群的神经毒性健康风险，并进一步利用网络毒理学和分子对接技术探究其潜在毒性机制。结果表明，宿舍区域目标化合物的含量最高，主要组分包括邻苯二甲酸二（2-乙基己基）酯、对苯二甲酸二辛酯、乙酰柠檬酸三丁酯及偏苯三酸三辛酯。暴露评估表明，经口摄入是人体暴露的主要途径。毒性当量（TEQ）分析显示，18～60岁女性面临的神经毒性风险高于男性。在机制上，网络毒理学筛选出59个核心靶点，主要富集于内分泌抵抗和癌症通路，表明这些化合物可能通过干扰细胞稳态与信号转导诱发神经毒性。分子对接识别出邻苯二甲酸二苯酯和邻苯二甲酸二环己酯为关键风险驱动因子，相比之下，芳香环和酯基较少的替代品（如己二酸庚基壬基酯、己二酸二异丁酯和己二酸二异癸烷基酯）表现出更低的神经健康风险和毒性潜力。构效关系分析表明，芳香环与酯基的协同作用是诱导神经毒性的关键机制。本研究通过整合环境暴露特征、TEQ评估与分子机制分析，揭示了高校特定人群的神经毒性风险特征，阐明了分子结构对神经毒性的影响机制，从而为低神经毒性替代品的定向筛选和室内环境健康风险管理提供了参考依据。

## Abstract (en)

Traditional phthalate esters （PAEs） and their novel alternatives are widely used as plasticizers. Owing to their non-covalent bonding with polymer matrices， these compounds readily migrate from materials and accumulate in indoor dust， posing potential risks to human health. Although their carcinogenic and reproductive toxicities have been extensively studied， their neurotoxicity， particularly that of novel alternatives， remains poorly understood. To address this knowledge gap， this study adopted an integrated approach combining pollution profiling， health risk assessment， and mechanistic investigation to systematically evaluate the neurotoxicity risks and potential mechanisms of PAEs and their alternatives in typical campus microenvironments （classrooms， laboratories， offices， cafeterias， and dormitories）. The contamination profile of target compounds in indoor dust was determined using comprehensive two-dimensional gas chromatography coupled with time-of-flight mass spectrometry （GC×GC-TOF MS）. By integrating three exposure pathways （ingestion， inhalation， and dermal contact of dust） with absorption， distribution， metabolism， excretion， and toxicity （ADMET） models， the estimated daily intakes （EDIs） and neurotoxic health risks for different populations were assessed. Furthermore， network toxicology and molecular docking techniques were employed to elucidate the potential toxic mechanisms. Results indicated that dormitories exhibited the highest contents of target compounds， with major components including di-2-ethylhexyl phthalate （DEHP）， di（2-ethylhexyl） tetrahydrophthalate （DEHTH）， acetyl tri-n-butyl citrate （ATBC）， and trioctyl trimellitate （TOTM）. Exposure assessment identified ingestion as the predominant exposure route. Using the ADMET model， toxicity equivalency factor （TEF） and toxic equivalent quantity （TEQ） were quantified for five neurotoxicity-related health endpoints， including phenotypic neurotoxicity， estrogen receptor activity， oxidative stress， mitochondrial dysfunction， and DNA damage. Risk assessment based on TEQ revealed that females aged 18-60 years faced higher neurotoxicity risks than males， although no statistically significant gender differences in EDI were observed across all age groups. Mechanistically， network toxicology identified 59 core targets associated with neurotoxicity， including oncogene， non-receptor tyrosine kinase （SRC）， serine/threonine kinase 1 （AKT1）， estrogen receptor 1 （ESR1）， mitogen-activated protein kinase （MAPK1， MAPK3）， heat shock protein 90 alpha family class a member 1 （HSP90AA1）， and Kirsten rat sarcoma viral oncogene homolog （KRAS）. Functional enrichment analysis showed that these core targets were predominantly enriched in pathways related to endocrine resistance and cancer， suggesting that these compounds may induce neurotoxicity by disrupting cellular homeostasis and signal transduction. Molecular docking supported specific binding interactions between representative compounds and core proteins， validating the predicted associations. Notably， diphenyl phthalate （DPhP） and dicyclohexyl phthalate （DCHP） were identified as the key risk drivers. In contrast， novel alternatives with fewer aromatic rings and ester groups， such as diheptyl， *N*-nonyl adipate （DHeNoA）， diisobutyl adipate （DiBA）， and diisodecyl adipate （DiDeA）， exhibited lower neurotoxic potential. Structure-activity relationship analysis suggested that the synergistic effect of aromatic rings and ester groups is a critical mechanism inducing neurotoxicity. By integrating environmental exposure profiling， TEQ-based risk assessment， and molecular mechanism analysis， this study not only delineates the neurotoxicity risk profile for specific campus populations but also elucidates the influence of molecular structure on neurotoxicity， providing a scientific basis for the targeted screening of low-neurotoxicity alternatives and informed risk management of indoor environmental health.

邻苯二甲酸酯（PAEs）作为增塑剂，被广泛用于食品包装、建筑材料和个人护理品中，以增强材料的柔韧性^［1，2］^。由于PAEs与聚合物基质主要通过非共价键结合，它们易于从产品中迁移并富集于室内环境（尤其是灰尘）中^［3］^，进而通过经口摄入、吸入及皮肤接触等途径进入人体，构成潜在的健康风险^［4］^。因此，美国、欧盟等地区相继出台了对PAEs的监管限制^［2］^，这导致PAEs替代品的广泛使用^［5］^。然而，随着替代品的普及，关于其在室内环境（尤其是灰尘）中的健康风险研究仍显滞后，其中针对神经毒性等特定健康终点的证据尤为有限^［6］^。

大多数人80%～90%的时间在室内度过^［4］^。室内灰尘作为污染物的关键暴露介质，被认为与人体健康尤其是神经系统不良反应密切相关^［7］^。作为室内灰尘中的主要污染物之一^［5］^，PAEs及其替代品引发的暴露风险与毒性机制受到广泛关注^［8］^。目前，大量研究已深入探讨了该类化合物的致癌性^［4，9］^、生殖毒性^［10］^、发育毒性^［11］^以及内分泌干扰效应^［12］^。相比之下，其神经毒性的研究则相对有限，且现有工作主要集中于传统PAEs，如邻苯二甲酸二（2-乙基己基）酯（DEHP）^［13］^和邻苯二甲酸二正丁酯（DBP）^［14］^，对新型替代品的关注严重不足。鉴于神经系统在生命早期发育中的关键作用及其对外界化合物暴露的高度敏感性，系统研究PAEs及其替代品的神经毒性十分必要。这不仅是对现有毒性知识体系的有效补充，也为全面的健康风险评估与防控策略的制定提供了重要参考。

传统毒理学方法因主要局限于单一终点评估，难以系统量化复合污染物的综合健康风险^［15］^。相比之下，效应导向分析可整合多源暴露数据，识别关键风险驱动因子并量化其健康贡献度，从而为污染物的优先管控提供科学依据^［16］^。值得注意的是，准确识别混合物中的关键毒物并评估其贡献，必须首先对潜在高风险化学物质本身的毒性机制有深入了解。因此，深入研究传统PAEs及其替代品的神经健康风险与潜在机制至关重要。其中，传统PAEs及其替代品的吸收（absorption）、分布（distribution）、代谢（metabolism）、排泄（excretion）与毒性（toxicity）特性（ADMET）是评估其神经健康风险的基础^［17］^。基于PAEs的化学结构，可以预测关键神经毒性终点，包括表型神经毒性、雌激素受体活性、氧化应激、线粒体功能紊乱及DNA损伤等^［14，18-21］^。这些毒性通路已被证实能够抑制神经前体细胞增殖、扰乱神经元的能量代谢和功能稳态^［19，20，22，23］^。然而，现有研究特别是针对新型替代品的部分，多停留在毒性效应表征层面，对其潜在作用机制的系统性阐释仍明显不足。与此同时，传统方法在揭示多靶点、多通路的复杂毒性机制方面存在一定局限。为克服这些不足，网络毒理学通过整合网络理论与系统生物学原理^［24］^，可构建生物相互作用网络以识别关键靶点和通路，从系统层面阐明毒性机制^［25］^；分子对接技术则能在分子水平上预测污染物与靶标蛋白的结合模式。两者协同，可在实验数据不足的条件下高效筛选PAEs及其替代品的神经毒性靶点并阐明分子机制^［15］^。

本研究旨在探究校园室内灰尘中传统PAEs及其替代品的神经毒性风险及潜在机制。基于课题组已建立的全二维气相色谱-飞行时间质谱联用（GC×GC-TOF MS）方法^［26］^，本研究对44种目标化合物（包括25种PAEs和19种替代品）进行分析。其中，替代品分为两类：①结构经优化但仍保留邻苯二甲酸酯骨架的新型PAEs；②不含邻苯二甲酸酯骨架但具有同等增塑功能的非PAEs类增塑剂。该成熟方法已在前期研究中系统验证，涵盖了PAEs及其替代品的标准品信息、样品采集与前处理标准化流程、仪器检测参数（色谱柱配置、温度程序、质谱条件等）和方法学性能指标（检出限、定量限、回收率和精密度）。基于此，本研究的主要目标包括：（i）解析室内灰尘中目标污染物的赋存特征（包括含量水平、组成谱与空间分布差异）；（ii）评估不同年龄和性别群体的暴露剂量及神经健康风险，为制定科学的风险管理策略提供依据；（iii）整合网络毒理学与分子对接技术，识别核心靶标并阐明其作用机制通路，为快速评估其潜在神经毒性及诊断相关病理提供科学见解。

## 1 实验部分

### 1.1 样品采集与前处理

2023年2月，在北京某高校的典型室内微环境中采集了163份灰尘样品。采样点涵盖实验室（*n*=55）、办公室（*n*=28）、宿舍（*n*=68）、教室（*n*=8）以及食堂（*n*=4），以全面评估校园人群的暴露场景。样品采集方法详见本课题组已建立的标准流程^［26］^。具体而言，将清洗后的尼龙采样袋作为吸尘器集尘腔的内衬，并使用该吸尘器采集地面灰尘。样品过150目筛去除大颗粒杂质，随后用铝箔包裹，于-20 ℃条件下避光保存。所得样品用于分析25种PAEs与19种替代品，各目标化合物的结构式见附图S1（www.chrom-China.com）。

### 1.2 每日暴露量评估

鉴于PAEs及其替代品在室内灰尘中的含量存在样本间空间差异与统计离散性（即变异性），为避免其通过室内灰尘的暴露风险被高估或低估，本研究采用蒙特卡洛模拟（Monte Carlo simulation， MCS）来更准确地评估经口摄入、吸入和皮肤接触3种途径的每日暴露量（estimated daily intake， EDI）。模拟使用Oracle Crystal Ball软件，对每种化合物进行10 000次迭代^［27］^。假设其含量（*C*_PAE，_ *_i_* ）服从正态分布，表示为（*μ*_PAE，_ *_i_* ，*σ*²_PAE_ *_，i_* ），其中*μ*_PAE，_ *_i_* 和*σ*_PAE，_ *_i_* 分别为平均含量（μg/g）和标准差，具体数值见附表S1。方差*σ* ^2^_PAE，_ *_i_* 由标准差的平方计算得到。

考虑到不同年龄群体EDI的差异，根据《中国人群暴露参数手册》将人群划分为8个年龄段：0～1岁、1～2岁、2～3岁、3～6岁、6～18岁、18～45岁、45～60岁和60岁以上^［28］^，并按性别分层。各暴露途径及总摄入量的EDI采用公式（1）～（4）进行计算，相关参数见附表S2。

EDI_ing=_
*C*_d_×*R*_ing_×EF/BW （1）

EDI_inh=_
*C*_d_×*R*_inh_×EF/(BW×R_PE_) （2）

EDI_der=_
*C*_d_×SA×ABF×AF×EF/BW （3）

EDI_total=_EDI_ing_+EDI_inh_+EDI_der_ （4）

其中，EDI_ing_、EDI_inh_、EDI_der_和EDI_total_分别表示通过灰尘经口摄入（ingestion）、吸入（inhalation）及皮肤接触途径（dermal absorption）和总体（total）的EDI（μg/（kg·day））；*C*_d_是室内灰尘中PAEs及其替代品的含量（μg/g）；*R*_ing_是每日摄入率；*R*_inh_是每日吸入率；EF是每日在室内环境中的暴露分数；BW是平均体重；*R*_PE_是颗粒物排放系数；SA是人体皮肤的暴露表面积；AF是灰尘在皮肤上的附着系数；ABF是皮肤的吸收分数。在此基础上，针对上述3种暴露途径，分别估算了不同年龄和性别组在中暴露（EDI-P50）和高暴露情景（EDI-P95）下的EDI^［1］^。

### 1.3 基于ADMET预测PAEs的神经毒性参数

ADMETlab 3.0是一款基于网络的在线预测平台，用于评估化合物的ADMET性质^［17］^。本研究采用PAEs及其替代品的毒性当量因子（toxicity equivalence factor， TEF）来评估其神经健康风险。TEF的核心作用在于量化各化合物在特定神经毒性终点上的相对效力，以便通过归一化处理进行风险加和。本研究选取了5个神经毒性相关终点：表型神经毒性（P-NT，指外源化学物对中枢及周围神经系统的整体损伤效应）以及4个神经毒性机制终点：雌激素受体活性（NR-ER）、氧化应激（SR-ARE）、线粒体功能（SR-MMP）和DNA损伤（SR-p53）。在中暴露水平下，化合物的毒性当量（TEQ， 单位为ng TEQ/（kg·day））由公式（5）计算得出，即EDI_total_与对应TEF的乘积，该值用于衡量其毒性潜力^［29］^。

TEQ=EDI_total_×TEF （5）

### 1.4 蛋白质-蛋白质相互作用网络构建与核心靶点识别

为阐明PAEs及其替代品的神经毒性机制，本研究整合了化学结构预测模型、网络分析算法与毒性评估方法。采用多种软件工具进行初筛，以获取其神经毒性相关信息。相关数据库及对应的统一资源定位符（URLs）详见附表S3。

首先，从PubChem数据库获取目标化合物的SMILE结构^［30］^，并利用SwissTargetPrediction平台（物种限定为“人类”）进行潜在靶点筛选^［31］^，共获得689个预测靶点。同时，以“神经毒性”“神经系统疾病”“神经损伤”为关键词，通过GeneCards和OMIM数据库检索与神经毒性相关的靶点，并以中位分值作为筛选阈值，最终获得10 917个神经毒性相关靶点。通过韦恩图分析，得到638个共同靶点作为候选靶点（附图S2）。随后，利用STRING平台构建上述候选靶点的蛋白质相互作用（protein-protein interaction， PPI）网络^［32］^，设定物种为“人类”且交互置信度大于0.9，得到一个包含159个节点和852条边的PPI网络图（平均节点度为10.72），具体靶点见附表S4。将该网络图导入Cytoscape软件（版本3.10.2）进行可视化^［33］^，并借助CytoNCA插件进行拓扑学分析^［34］^。通过设定介数中心性（betweenness centrality， BC）、接近中心性（closeness centrality， CC）和度值3个参数的中位数为阈值^［25］^，筛选出59个核心靶点，并绘制核心PPI网络图（附图S3），揭示关键靶点之间的互作模式。其中，节点度值最高的7个核心靶点分别为原癌基因非受体酪氨酸激酶（oncogene， non-receptor tyrosine kinase，SRC）、丝氨酸/苏氨酸激酶1（serine/threonine kinase 1，AKT1）、雌激素受体1（estrogen receptor 1，ESR1）、丝裂原活化蛋白激酶1（mitogen-activated protein kinase 1， MAPK1）、丝裂原活化蛋白激酶3（mitogen-activated protein kinase 3， MAPK3）、热休克蛋白90α家族A类成员1（heat shock protein 90 alpha family class a member 1， HSP90AA1）、Kirsten大鼠肉瘤病毒癌基因同源物（Kirsten rat sarcoma viral oncogene homolog， KRAS，一种编码GTP酶的关键原癌基因）。

### 1.5 功能富集与通路分析

为进一步阐明PAEs及其替代品所致神经毒性核心靶点的生物学功能，本研究利用DAVID平台对上述关键靶点进行了基因本体论（Gene Ontology， GO）和京都基因与基因组百科全书（Kyoto Encyclopedia of Genes and Genomes， KEGG）通路富集分析^［35］^。GO分析涵盖3个基本的生物学维度：分子功能（molecular functions， MF）、生物过程（biological processes， BP）与细胞组分（cellular components， CC）。KEGG通路分析则用于识别与核心靶点相关的关键信号通路^［36］^。分析物种设定为“人类”，以*p<*0.05为显著性阈值，并对结果按*p*值进行排序。最终，选取GO各维度（MF、BP、CC）前10项条目及KEGG前20条通路进行可视化，同时使用Cytoscape构建化合物、核心靶点与主要KEGG通路相互作用网络。

### 1.6 PAEs及其替代品与核心靶点的分子对接

本研究采用分子对接技术，以预测PAEs及其替代品与核心靶点的结合构象与亲和力，从而揭示它们之间的分子相互作用机制^［36］^。具体而言，首先，从PubChem数据库获取PAEs及其替代品的SDF格式文件，并使用ChemDraw3D软件将其转换为PDB格式^［24］^。随后，将PDB格式的化合物导入AutoDock 1.5.7软件，并转换为PDBQT格式。同时，从蛋白质数据库（Protein Data Bank， PDB）下载靶点蛋白的三维结构（PDB格式），利用Pymol 2.5.0去除蛋白结构中的水分子和原始配体。接着，将处理后的蛋白导入AutoDock进行加氢、合并非极性氢原子与计算电荷分配，最终保存为PDBQT格式的受体文件。而后，使用AutoDock vina 4.2对核心靶蛋白和化合物进行分子对接，并评估其结合自由能。最后，结合模式和分子相互作用在PyMOL中可视化^［36］^。为验证对接结果的可靠性，本研究将核心靶点与其内源性共晶配体进行重新对接，并通过计算共晶配体构象与预测结合位点之间的均方根偏差（root-mean-square deviation， RMSD）进行评估。

## 2 结果与讨论

### 2.1 室内灰尘中PAEs及其替代品的含量与分布

在检测的44种化合物中，共有24种化合物的检出率（detection frequency， DF）≥50%，被纳入后续分析，其中DF、平均含量及占比详见附表S5。总体来看，宿舍中Σ_24_PAEs平均含量（356.52 μg/g）显著高于其他功能区（218.98～260.66 μg/g）（图1a）。进一步根据平均含量，将24种化合物分为高、中、低3个丰度类别，其分布总体呈现“高丰度优势物种与中低丰度广泛共存”的复合污染特征。暴露水平与物种组成均受功能区使用特征和材料属性的共同驱动，表明暴露风险具有场所特异性。

**图1:**
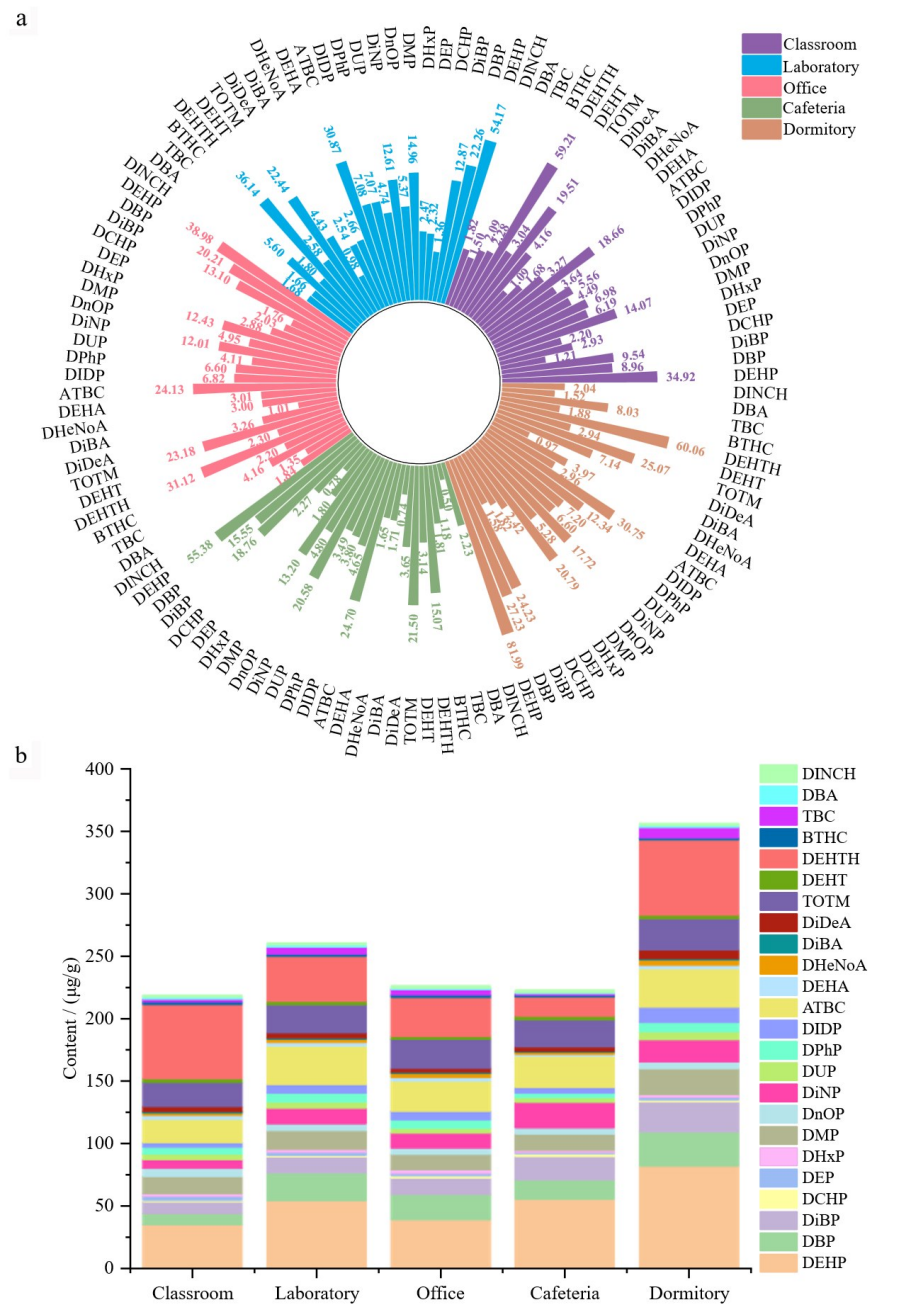
不同校园微环境室内灰尘中主要PAEs及其替代品组分的（a）平均含量比较和（b）含量堆积图

高丰度化合物（各功能区平均含量均超过15 μg/g）包括DEHP、对苯二甲酸二辛酯（DEHTH）、乙酰柠檬酸三丁酯（ATBC）和偏苯三酸三辛酯（TOTM）（图1b）。其中，DEHP普遍检出且含量较高，这可能与其产量大、用途广^［37］^及低蒸气压^［38］^有关。作为DEHP的主要替代品^［1］^，DEHTH的高含量可能与DEHP限用后其用量增加有关^［2］^。ATBC的大规模生产（年产量10 000～100 000 t）^［39］^及其向灰尘介质的高释放潜力（79.80 mg/kg）^［15］^，共同导致该物质易于从聚合物材料中迁移并在室内灰尘中累积。相比之下，TOTM虽不易从产品中迁移至灰尘，但一旦进入，其极低的挥发性和优异的热稳定性可显著抑制二次挥发^［40］^，从而在室内环境中表现出更高的滞留性和累积潜力。

中等丰度化合物（5～15 μg/g）包括DBP、邻苯二甲酸二异丁酯（DiBP）、邻苯二甲酸二甲酯（DMP）和邻苯二甲酸二异壬酯（DiNP）。这些化合物在所有功能区普遍存在，但含量呈现显著的空间分异，尤其以宿舍的富集最为突出。

低丰度化合物（<5 μg/g）包括邻苯二甲酸二异癸酯（DiDP）、邻苯二甲酸二苯酯（DPhP）、邻苯二甲酸二正辛酯（DnOP）和邻苯二甲酸二环己酯（DCHP）等。这些化合物虽然检出率高但含量较低，呈现典型的“低剂量，广泛分布”特征。

本研究进一步分析了室内PAEs及其替代品在不同微环境中的含量差异及成分特征。宿舍灰尘中DEHP含量（81.99 μg/g）约为教室（34.92 μg/g）和办公室（38.98 μg/g）的2～3倍，这可能与DEHP在个人护理用品（如面霜和身体乳）^［41］^、软质家具（如床垫）^［42］^以及纺织品（如衣物）^［43］^中的广泛使用有关。宿舍中DBP含量（27.23 μg/g）约为教室（8.96 μg/g）的3倍，该差异亦可能源于个人护理品及纺织品的频繁使用^［43，44］^。除使用源特征外，宿舍通风不良可能进一步加剧污染物的累积。教室灰尘中DEHTH含量（59.21 μg/g）约为食堂（15.07 μg/g）的4倍。这一差异主要受释放源强度与通风清除效率的共同影响。在教室中，地板、涂料和电子设备等构成了强度较高且持续的DEHTH释放源^［1，45］^；同时，DEHTH挥发性较低，加之教室通风条件通常欠佳，从源头释放出的DEHTH难以有效扩散，从而更易吸附并富集于灰尘中，导致持续累积。相比之下，食堂的情况则有所不同：烹饪活动导致环境温度较高，从而在一定程度上促进了DEHTH的释放^［46］^；然而，其通风系统效果显著，能够迅速将空气中的DEHTH排出，从而限制其在灰尘中的沉降与累积。总体而言，室内灰尘中PAEs的成分差异受到建筑结构、应用范围及监管限制等多种因素的影响^［9］^。

### 2.2 人体经室内灰尘每日暴露于PAEs及其替代品的水平

本研究基于高校环境采集的室内灰尘样本，评估了PAEs及其替代品的EDI。需要特别指出的是，鉴于采样环境特定于高校，其灰尘成分及含量特征可能不同于家庭、幼儿园等其他生活场所。因此，基于高校灰尘数据对非高校活动人群（尤其是婴幼儿）的暴露水平进行推算时，可能存在不确定性。本研究结果主要反映在给定模型参数与高校灰尘含量条件下的相对风险趋势。在不同人群的暴露估算中，附表S6展示了按年龄和性别划分，在EDI-P50和EDI-P95情景下，经EDI_ing_、EDI_inh_、EDI_der_和EDI_total_途径接触PAEs及其替代品的估算值。在中暴露情境下，EDI_total_范围为0.183～1.536 μg/（kg·day），主要暴露物质为DPhP，其次是ATBC和邻苯二甲酸二乙酯（DEP）。经口摄入暴露量比吸入高3到4个数量级，比皮肤吸收暴露高2到3个数量级，表明经口摄入为主要的暴露途径（图2）。该结果与已有研究一致^［37，47］^，这归因于PAEs易从产品中迁移至灰尘中^［48］^，并可能通过手口行为被摄入。此外，由于PAEs的皮肤吸收率极低（0.000 031～0.000 778），皮肤吸收途径的影响可忽略不计^［1］^。高相对分子质量PAEs（如TOTM、DEHP、ATBC等）挥发性较低，这也可能是导致吸入途径暴露贡献较小的原因之一^［48］^。尽管皮肤吸收与吸入途径的暴露风险相对较低，其长期健康影响仍值得关注。在高暴露情境下，也观察到相似的暴露趋势。

**图2:**
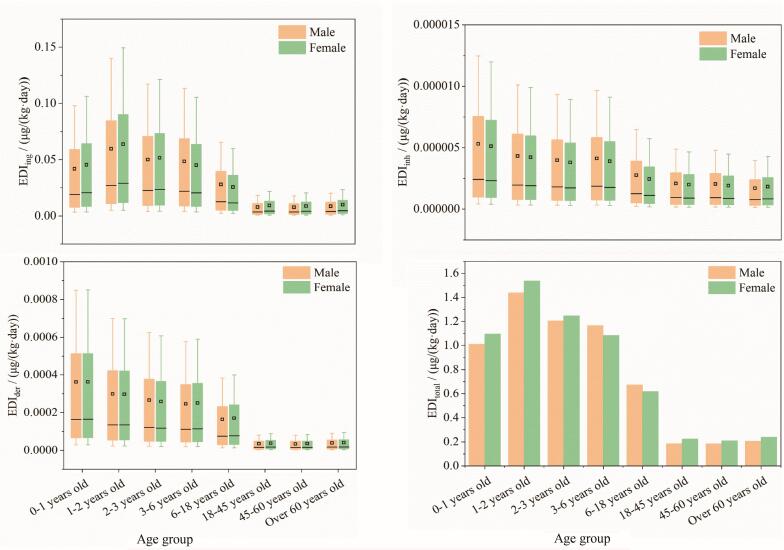
在中暴露情景下，室内灰尘中PAEs及其替代品通过不同途径估计的每日摄入量

此外，作为对不同人群暴露差异的补充参考，婴幼儿组显示出更高的暴露风险，这与其频繁的手口接触行为、较低的体重及较长的暴露时间等特征相符^［47］^。但需指出，考虑到本研究的采样环境限定于高校，婴幼儿并非该环境中的主要暴露群体，因此其风险评估结果主要用于跨人群暴露敏感性对比。实际的健康风险评估应更聚焦于高校环境的主要暴露人群（18～60岁），包括学生、教职工及后勤服务人员。结果显示，18～60岁群体的EDI_total_值虽低于婴幼儿，但仍表明室内灰尘中的PAEs及其替代品对该群体存在潜在健康风险，值得进一步关注。在所有年龄组中，中暴露情景下不同性别的EDI值无显著差异（*p*>0.05，Mann-Whitney U test）。值得注意的是，男性的EDI_inh_值相对较高，这可能由生理差异与行为因素共同驱动。在生理层面，男性通常具有更高的呼吸参数（如肺活量、每分钟通气量），导致在相同灰尘含量环境下单位时间的潜在吸入量更大；在行为层面，个体的活动模式（如室内活动频率与活动强度）可能是潜在的影响因素，但其具体驱动机制及贡献度有待通过行为学观测等研究进一步量化。

### 2.3 PAEs及其替代品的神经毒性暴露风险评估

利用ADMETlab平台预测了PAEs及其替代品在5种神经毒性终点上的TEF（附表S7）。基于TEF和EDI-P50进一步计算了TEQ（附表S8），以系统评估不同人群的神经毒性健康风险。为更精准聚焦高校环境，本研究选取了3个功能互补的关键群体开展TEQ分析：（1）18～45岁女性，覆盖实际采样的主要群体，反映高校环境下成年人的主导暴露水平及健康风险；（2）6～18岁男性，代表处于发育关键期的性别差异群体，兼顾青春期暴露特征及高校潜在人群（如高中生及准大学生群体）；（3）1～2岁女孩，作为理论上暴露风险最高且生理最敏感群体，用于界定参数敏感性风险边界。其余群体的风险变化趋势可由上述3类代表性群体所覆盖（图3）。结果显示，在高校主要暴露群体（18～45岁）中，女性的TEQ值高于同龄男性，提示该群体在高校特定环境下存在不可忽视的神经毒性风险。6～18岁男性群体的TEQ值处于较高水平，说明其暴露差异可能同时受EDI暴露量和生理特征共同驱动。1～2岁女孩的TEQ值最高，与其较高的灰尘摄入量和生理敏感性相符。然而，鉴于本研究样本来源于高校环境，该结果更适合作为跨人群敏感性比较的参考依据。单体贡献分析表明，DPhP是所有年龄段TEQ的主要驱动因子，尤其在表型神经毒性、雌激素受体活性和线粒体功能3个关键终点上均表现出最高的TEF和TEQ值；DCHP则在除雌激素受体活性外的其他神经毒性终点中贡献较高。综上所述，尽管婴幼儿群体暴露风险最高，本研究核心关注点仍为高校环境中成年人的暴露风险。尤其18～60岁女性群体表现出较高的神经毒性风险，应作为高校健康风险管控的优先关注对象。本研究结果可为高校环境暴露风险识别、场所健康管理及精准干预提供科学依据。

**图3:**
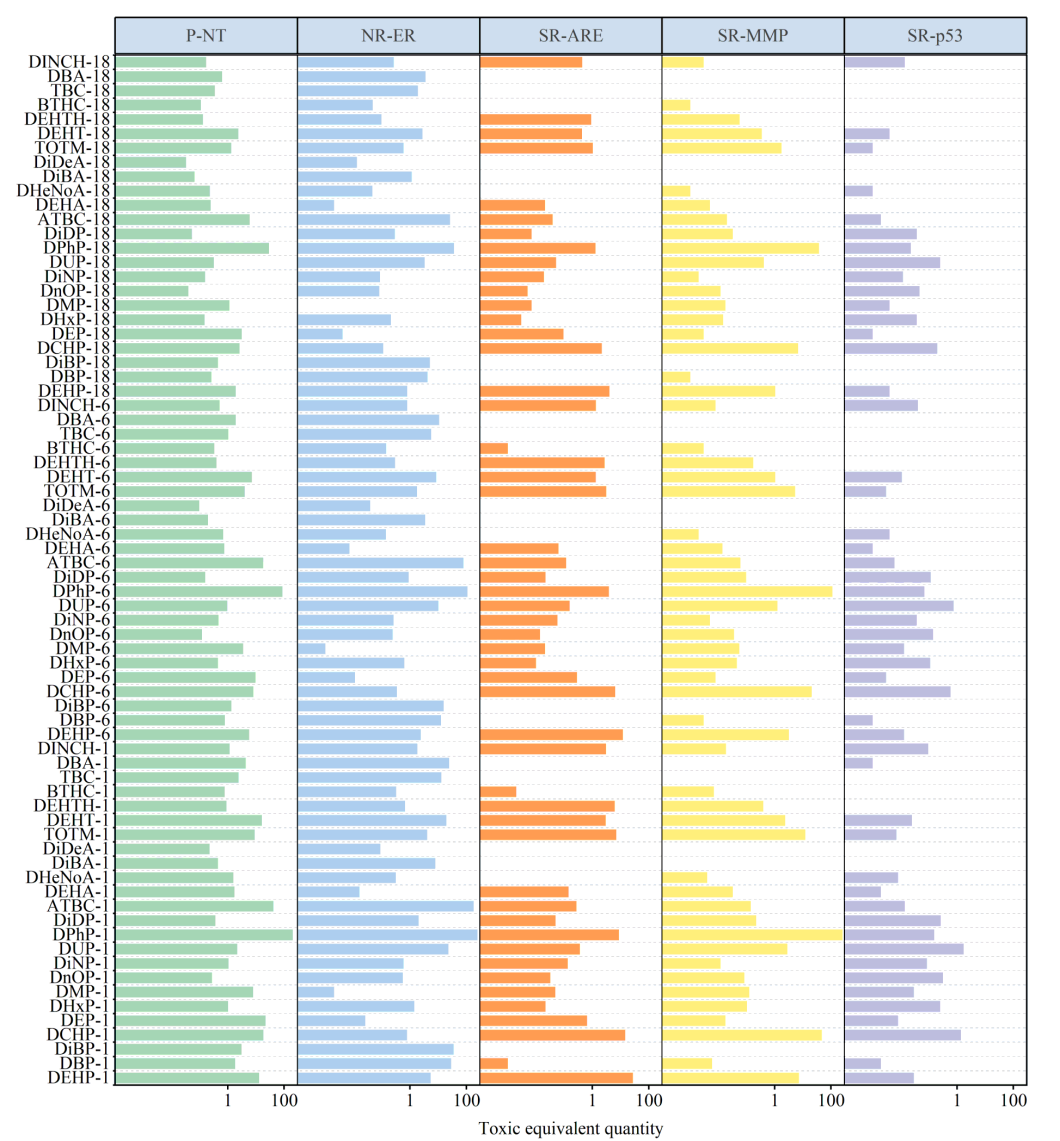
1～2岁女孩、6～18岁男孩和18～45岁女性中PAEs及其替代品的毒性当量

在表型神经毒性方面，DPhP（毒性当量29.40 ng TEQ/（kg·day））、ATBC（毒性当量6.09 ng TEQ/（kg·day））、DEP（毒性当量3.20 ng TEQ/（kg·day））、DCHP（毒性当量2.69 ng TEQ/（kg·day））、二（2-乙基己基）四氢邻苯二甲酸酯（DEHT，毒性当量2.40 ng TEQ/（kg·day））和DEHP（毒性当量1.92 ng TEQ/（kg·day））等化合物贡献显著。该结果印证了神经系统是PAEs及其替代品的关键易感靶标^［14］^。其神经毒性机制已在多个研究层面得到验证：在分子层面，DEHP可穿过血脑屏障直接损伤神经元，这为其影响认知和行为提供了生理基础^［49］^；在动物模型中，DEHP在秀丽隐杆线虫中可诱发身体弯曲、头部跳跃等异常行为^［13］^。DEP和ATBC则通过抑制斑马鱼乙酰胆碱酯酶活性并改变神经发育相关基因的表达，从而干扰其正常的神经系统发育^［14，50］^；在人群流行病学层面，DEHP暴露与认知障碍和孤独症谱系障碍的风险增加有关^［51］^。

在干扰雌激素受体活性方面，DPhP（毒性当量36.25 ng TEQ/（kg·day））、ATBC（毒性当量26.51 ng TEQ/（kg·day））、DiBP（毒性当量5.17 ng TEQ/（kg·day））和DBP（毒性当量4.19 ng TEQ/（kg·day））是主要贡献者。DBP已被证实可通过激活雌激素受体，减少斑马鱼大脑中增殖神经元的数量，并干扰相关受体基因（esr1、esr2a和esr2b）的表达^［22］^。此外，PAEs还可能通过影响雌激素的合成，间接损害神经发育^［18］^。

在氧化应激方面，DEHP（毒性当量4.03 ng TEQ/（kg·day））、DCHP（毒性当量2.17 ng TEQ/（kg·day））和DPhP（毒性当量1.28 ng TEQ/（kg·day））是主要的贡献化合物。这一毒性通路至关重要，因为氧化应激在神经退行性疾病的病理过程中扮演着核心角色^［19］^。多项研究为此提供了直接证据：DEHP暴露不仅可升高小鼠脑组织的氧化应激水平，还会损害其后代的空间记忆能力^［13］^；长期接触DEHP还会导致谷胱甘肽生物合成途径失调，最终导致神经行为与神经发育异常^［52］^；DCHP与DPhP也显示出诱导氧化应激的潜力，前者在银鱼卵黄中的生物蓄积可诱发跨代氧化应激效应^［53］^，而两者均能破坏鲫鱼体内的氧化还原平衡^［54］^。

在线粒体功能障碍方面，DPhP（毒性当量37.62 ng TEQ/（kg·day））、DCHP（毒性当量6.87 ng TEQ/（kg·day））、TOTM（毒性当量1.77 ng TEQ/（kg·day））和DEHP（毒性当量1.06 ng TEQ/（kg·day））共同构成核心风险。例如，DEHP可通过破坏内质网与线粒体的结构和信号互作诱发神经毒性^［8］^。鉴于线粒体在神经元能量代谢、氧化还原平衡及钙稳态维持中的核心作用，其功能受损被认为是PAEs及其替代品介导神经元损伤的关键机制之一^［20］^。

在DNA损伤方面，邻苯二甲酸二十二烷基酯（DUP，毒性当量0.25 ng TEQ/（kg·day））、DCHP（毒性当量0.20 ng TEQ/（kg·day））和DnOP（毒性当量0.05 ng TEQ/（kg·day））被识别为潜在的风险物质。其中，DnOP可导致蚯蚓DNA损伤，且在高剂量下效应更为显著^［55］^。这类损伤的潜在后果值得警惕，因为DNA损伤的积累会激活p53等通路，进而调控细胞凋亡与周期阻滞^［56］^，并可能最终诱发神经退行性病变^［21］^。

综上所述，基于TEQ的评估表明，DPhP、DCHP和DEHP在表型神经毒性、氧化应激及线粒体功能障碍等多个神经毒性相关终点中均表现出较高的暴露贡献值，显示出其具有广泛的毒性潜力。其中，DPhP在干扰雌激素受体活性方面贡献最为突出，而DCHP则在诱导DNA损伤方面优势明显。尽管本研究结果尚不能直接推断具体健康风险，但明确指出DPhP和DCHP在多个神经毒性机制中具有更高的暴露潜在性，值得后续毒理学研究中重点关注。此外，PAEs及其替代品的神经毒性机制十分复杂，可能是多种毒性通路联合作用的结果^［13，56，57］^。

### 2.4 PAEs及其替代品诱导神经毒性的潜在机制

#### 2.4.1 核心靶点的GO和KEGG富集分析

基于DAVID数据库，我们对59个核心靶点进行了GO功能注释与KEGG通路富集分析。GO分析共产生587个具有统计学显著性的条目，包括428个BP、59个CC和100个MF。所有GO条目（附表S9）已按*p*值排序。为进一步展示结果，我们从BP、CC和MF中分别筛选出*p*值最低的前10个条目进行可视化（图4a）。KEGG通路分析共识别出166条显著富集通路，其中*p*值最小的前20条通路被展示于图4b。

**图4:**
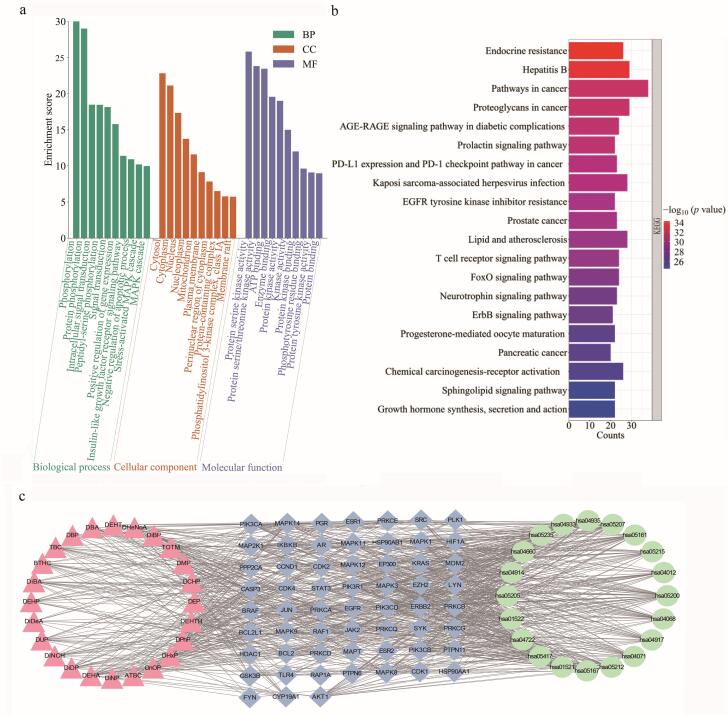
PAEs及其替代品诱导神经毒性的分子机制

分析结果表明，核心基因功能分布广泛，主要涉及神经信号传递、细胞信号转导和程序性细胞死亡等过程。关键KEGG通路分析显示，目标物的神经毒性可能源于多重机制的协同作用：其一，通过“内分泌抵抗”和“激素反应”通路干扰神经内分泌稳态；其二，通过“癌症相关通路”与“信号转导”通路扰乱调控神经元存活与死亡的核心信号网络；其三，与“乙型肝炎”通路所提示的神经免疫炎症机制相关。综上，目标物可能通过上述关键通路构成的复杂调控网络，共同影响神经相关生物学过程，最终诱发神经毒性。

#### 2.4.2 关键核心靶点的功能阐释

为阐释从PPI网络中筛选出的7个关键核心靶点（SRC、AKT1、ESR1、MAPK1、MAPK3、HSP90AA1、KRAS）在神经毒性中的作用，我们进行了系统的文献调研。结果表明，这些靶点共同构成了一个调控神经系统稳态的核心功能网络，其功能障碍是多种神经病理过程的共同基础。这些靶点的功能主要汇聚于以下几个关键方面：在突触功能与信号转导方面，SRC、MAPK1和MAPK3等在神经元分化、突触可塑性及信号传导中发挥核心作用；例如，SRC可直接调控神经元与突触活动^［58-60］^；在细胞存活与死亡调控方面，AKT1和KRAS是关键的信号节点，共同调控细胞的存活与凋亡进程。其中，KRAS更是AD中神经元细胞周期异常重启的关键驱动因子^［61-64］^；在神经免疫与蛋白稳态方面，HSP90AA1不仅作为免疫调节的分子枢纽^［65］^，其功能失衡还会破坏突触稳态，并加剧β-淀粉样蛋白（Aβ）的病理过程^［66］^；在基因表达与神经内分泌调控方面，ESR1通过调控大脑基因表达和神经发生，对维持高级认知功能至关重要^［67，68］^。

综上所述，本研究的结果提示，PAEs及其替代品的神经毒性可能并非通过单一靶点，而是通过协同干扰上述核心靶点所构成的调控网络来实现的。该网络的功能紊乱会共同导致突触功能障碍、神经元死亡、神经免疫失衡以及基因表达异常等一系列病理变化，最终引发神经毒性效应。此发现为揭示PAEs神经毒性的分子机制提供了一个全新的、系统性的视角。

#### 2.4.3 PAEs及其替代品诱导神经毒性的网络分析

使用Cytoscape构建了化合物、靶点和通路的相互作用网络，以系统探究其潜在的神经毒性机制（图4c）。结合网络分析结果与生物学功能相关性，我们筛选出4条关键通路进行深入阐释。首先，内分泌抵抗通路（hsa01522）的富集提示环境化合物可能作为内分泌干扰物通过该通路介导神经毒性^［69］^。具体而言，此类化合物可引发激素信号紊乱，特别是导致性激素水平降低；研究证实这与AD、PD和认知障碍的风险增加相关^［67，70，71］^，并伴随神经元与突触的形态改变^［70］^。其次，癌症通路（hsa05200）的富集揭示了其在神经毒性中的重要作用。该通路汇聚了大量调控细胞周期、凋亡与DNA修复的核心信号分子。研究表明，该通路在外周肿瘤中的异常激活可通过破坏血脑屏障完整性、诱发中枢免疫细胞浸润等机制，导致焦虑、抑郁及认知功能障碍^［72］^。据此推测，相关化合物可能通过干扰该通路中的关键靶点，破坏神经元的存活与内环境稳态。第三，神经营养素信号通路（hsa04722）是直接调控神经元发育、存活与凋亡的核心通路^［73］^。该通路通过神经营养因子与Trk家族酪氨酸激酶受体或p75神经营养素受体（p75NTR）的结合，精确调控神经元的迁移、分化、突触可塑性^［74］^，其功能失调已被广泛证实与PD、AD、癫痫等多种神经系统疾病的病理过程密切相关^［73］^。最后，FoxO信号通路（hsa04068）作为细胞应激反应的关键调控者，在决定神经元命运和维持中枢神经系统稳态中扮演核心角色。该通路通过响应氧化应激等刺激，参与调控神经元的自噬与凋亡过程，其失衡是神经退行性疾病发生发展的重要机制之一^［75］^。综上所述，这4条通路分别从内分泌干扰、细胞稳态失衡、神经营养支持中断和应激反应失调等多个互补的生物学层面，共同揭示了化合物诱导神经毒性的系统性机制，为后续实验验证与机制研究提供了明确的理论依据。

进一步的网络分析显示，上述通路与多个核心靶点存在紧密关联。hsa01522、hsa05200、hsa04722和hsa04068通路与核心靶点AKT1、MAPK1、MAPK3及KRAS密切相关。其中，hsa01522通路显著关联SRC，而hsa05200通路主要涉及HSP90AA1和ESR1。在化合物层面，DUP与HSP90AA1/ESR1、DnOP与KRAS、己二酸二2-乙基己基酯（DEHA）与SRC/AKT1以及TOTM与MAPK1/MAPK3之间的相关性，表明这些化合物可能具有显著的神经毒性。此外，DPhP、DCHP和DEHP与MAPK信号通路中的MAPK8、MAPK11和MAPK14相关。这些特异性关联共同提示，上述核心靶点极有可能是介导神经毒性的关键分子受体，值得在后续研究中开展深入的功能验证。

### 2.5 PAEs及其替代品与关键靶点的分子对接

基于网络毒理学分析结果，我们对筛选出的化合物与7个核心靶蛋白（SRC、AKT1、ESR1、MAPK1、MAPK3、HSP90AA1和KRAS）进行分子对接，以评估其相互作用。附表S10列出了各蛋白质结合口袋的位置和网格框尺寸。根据附表S11所列数据，所有化合物与靶蛋白在最佳结合构象下的结合能均低于-20.92 kJ/mol （-5 kcal/mol），表明二者间存在较强的亲和力和稳定的结合构象^［36］^。为验证对接方法的可靠性，我们从晶体结构中提取原始共结晶配体，并重新对接到相应蛋白活性口袋中（附图S4）。结果显示，对接构象与原始晶体构象高度吻合，其RMSD值均小于2 Å，符合分子对接可靠性判断标准，证实了本研究对接流程与结果的可靠性^［76］^。上述对接结果表明，PAEs及其替代品能够自发地与核心靶点结合，这可能在神经毒性机制中发挥关键作用。

化合物与核心蛋白之间的结合主要依靠氢键、范德华力、*π*-σ、*π*-烷基及*π*-*π*堆叠等多种非共价键相互作用来稳定。结构-活性关系分析显示，芳香环和酯基的数量是影响其与受体结合强度和毒性差异的主要因素。其中，芳香环主要通过*π*-σ、*π*-烷基和*π*-*π*堆叠作用参与疏水环境和空间稳定；而酯基则常作为氢键受体，通过形成氢键来增强结合特异性与稳定性。结合能最低的6个复合物为DPhP-HSP90AA1、DCHP-HSP90AA1、DPhP-AKT1、DCHP-AKT1、DPhP-KRAS，和DCHP-ESR1（见图5），其结合能分别为-42.94、-40.66、-40.52、-39.96、-38.89和-38.32 kJ/mol（依次对应为-10.263、-9.717、-9.685、-9.551、-9.295和-9.159 kcal/mol）。该结果明确表示，DPhP和DCHP与靶蛋白具有最强的结合亲和力。这一发现与健康风险评估结果相互印证：尽管室内灰尘中DPhP和DCHP的含量低于DEHP、DEHTH、ATBC和TOTM，但其更强的结合能力可能导致计算得到的健康风险水平更高，这可能与其分子结构中较多的芳香环有关。同时，高含量的DEHP和TOTM也表现出较强的结合力，需关注其神经毒性风险。相比之下，芳香环和酯基数量较少的替代品，如己二酸庚基壬基酯（DHeNoA）、己二酸二异丁酯（DiBA）和己二酸二异癸烷基酯（DiDeA），则表现出较低的结合亲和力与潜在风险。这些发现为开发神经毒性风险更低的新型替代品提供了结构设计方向。

**图5:**
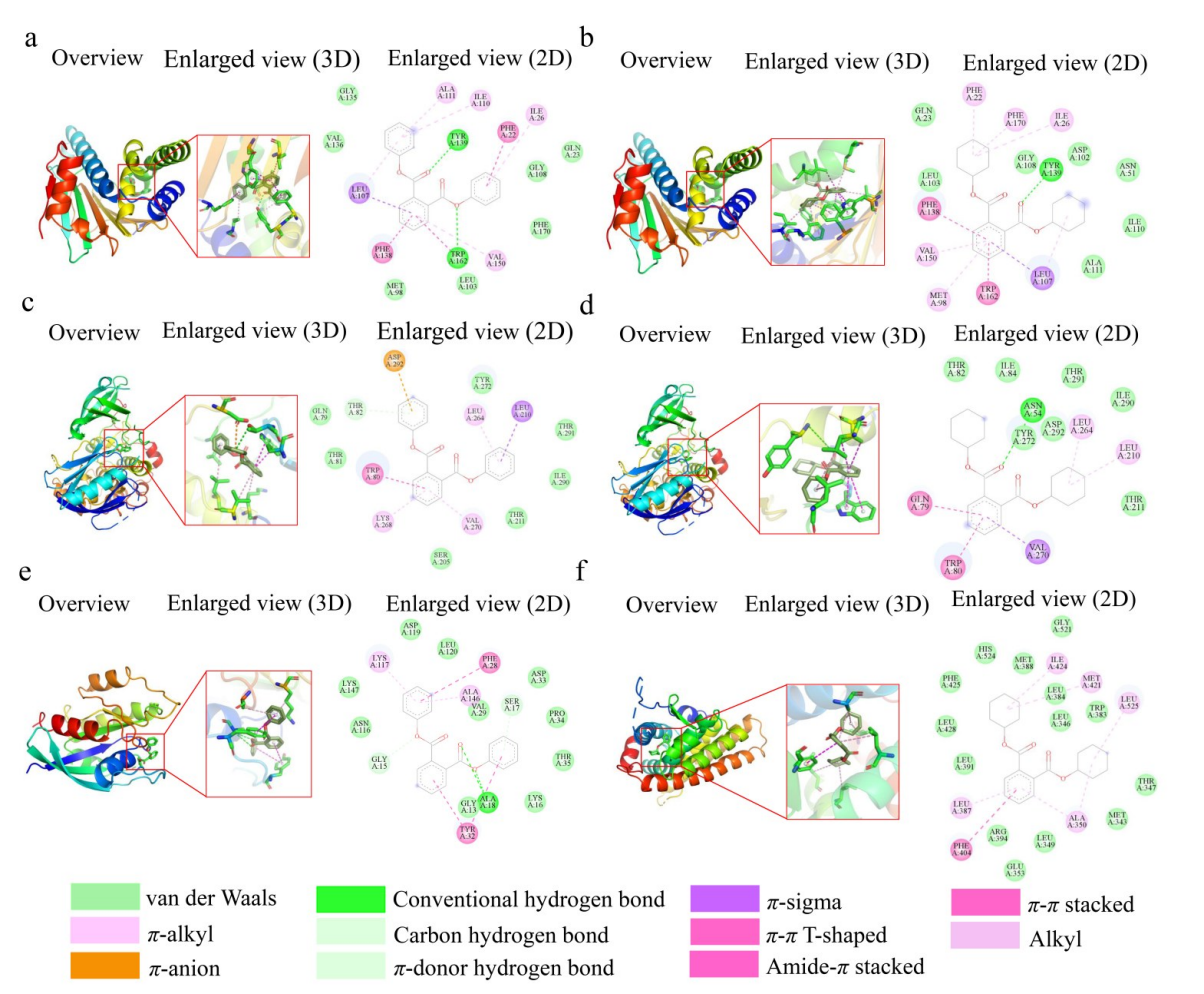
PAEs及其替代品与各靶蛋白分子对接的最佳结合构象

## 3 结论

本研究结合成分分析、健康风险评估、网络毒理学和分子对接技术，系统评估了校园室内灰尘中PAEs及其替代品的神经毒性暴露风险和潜在机制。结果显示，DEHP、DEHTH、ATBC和TOTM是室内灰尘中的主要污染物，其中宿舍区域的含量最高。暴露评估表明，经口摄入是人体暴露的主要途径。利用ADMET数据库，我们还评估了与神经暴露风险相关健康终点的TEQ。结果表明，18～60岁女性面临的神经毒性风险高于男性。进一步通过网络毒理学和分子对接技术，初步识别了关键靶点和信号通路，并探讨了其潜在的神经毒性机制。研究发现，DPhP和DCHP在多种神经毒性相关终点中表现出较高的暴露贡献，提示二者为值得关注的潜在神经毒性物质。总体而言，本研究为理解室内灰尘中PAEs及其替代品的潜在神经毒性风险提供了基础数据，也为更安全替代品的筛选和室内污染物健康风险防控策略的制定提供了参考依据。

然而，本研究仍存在一定局限性。首先，在暴露评估中，我们基于环境含量与暴露参数计算了外暴露剂量，但未纳入化合物在体内的毒代动力学过程（如吸收、分布、代谢和排泄），这可能影响对实际抵达靶器官的内暴露剂量及神经毒性风险的精确评估。其次，尽管通过网络毒理学与分子对接预测了化合物的神经毒性机制，这些预测结果尚未在相关的体内外生物模型中得到实验验证。因此，未来的研究有必要在明确毒代动力学参数的基础上，借助神经元或脑类器官等实验模型，对本研究筛选出的关键化合物（如DPhP和DCHP）及其与SRC、AKT1等核心靶点的相互作用进行机制验证，这将为神经毒性风险评价提供更坚实的实验依据。
